# Social Prescribing in Portugal: Study Protocol for a Primary Care Intervention Addressing Loneliness and Well-Being

**DOI:** 10.7759/cureus.101257

**Published:** 2026-01-10

**Authors:** Gladys L Caldeira, Beatriz Benquerenca, Laura Sequeira, Ana Rita Magalhães, Joana Penetra, Carolina Figueiredo, Rita A Fonseca, Mariana Trindade, Margarida Carmo, Ana I Campos, Maria João D Barbosa, Ana Filipa F Santos, Catarina Silva, Cristina Neves, Adriana Ventura, Susana Miguel, Inês Rosendo

**Affiliations:** 1 Primary Healthcare, Unidade de Saúde Familiar (USF) Topázio, Unidade Local de Saúde (ULS) Coimbra, Coimbra, PRT; 2 Medicine, Universidade de Coimbra, Coimbra, PRT; 3 Primary Healthcare, Unidade de Saúde Familiar (USF) Coimbra Norte, Unidade Local de Saúde (ULS) Coimbra, Coimbra, PRT; 4 Primary Healthcare, Unidade de Saúde Familiar (USF) Coimbra Centro, Unidade Local de Saúde (ULS) Coimbra, Coimbra, PRT

**Keywords:** family medicine, loneliness, social isolation, social prescription, well-being

## Abstract

Loneliness is one of the most relevant social health determinants in the elderly population, associated with polypharmacy, increased use of health services, cognitive decline, and increased morbidity and mortality. In Portugal, the high prevalence of loneliness among older adults highlights the urgent need for innovative strategies that go beyond conventional responses based mainly on biomedical approaches. Social prescribing (SP) is a promising non-pharmacological intervention characterized by the formal, structured referral of patients to community activities tailored to their needs, interests, and life context. International experiences, particularly in the United Kingdom, have informed the development and implementation of social prescribing initiatives across healthcare settings. However, the literature identifies limitations, including heterogeneity in recruitment methodologies, a scarcity of long-term studies, limited integration of sociocultural variables, and the absence of robust frameworks for evaluating and monitoring results.

In this context, this project aims to implement and evaluate a pilot model of SP in primary healthcare (PHC) for people aged 65 years or over, monitored in three family health units (FHUs) in central Portugal. The main objective is to evaluate the impact of the intervention on participants' perceptions of loneliness and well-being. Additionally, this study aims to identify individual and contextual factors that influence adherence, as well as barriers and facilitators, and to evaluate the implementation process in a real PHC setting. The design is prospective and longitudinal, and it integrates quantitative and qualitative assessments. The intervention involves recruiting users via the ALONE and Personal Well-Being Index (PWI) scales and SP appointments, during which, through shared decision-making, community-based cultural, recreational, sports, and/or social activities are prescribed. The evaluation and monitoring of outcomes will be based on validated scales (ALONE, PWI, UCLA, EQ-5D, and PHQ-4) administered at baseline and at three, six, 12, and 18 months. Additionally, the implementation will be evaluated by analyzing barriers and facilitators, as well as professionals' and participants' perceptions, using the RE-AIM framework. We plan to recruit 165 participants to ensure adequate statistical power, given possible follow-up losses. Expected results include a reduction in perceived loneliness, improved well-being and quality of life, characterization of adherence determinants, and recommendations for the sustainable integration of SP into PHC. This pilot study has the potential to generate innovative and transferable knowledge that supports the consolidation of the National Social Prescription Network and the formulation of person-centered health policies. By integrating health and community, it seeks to promote active aging, well-being, and social inclusion, reinforcing the PHC's role as a privileged space for innovative and cost-effective responses to the social determinants of health.

## Introduction

Loneliness is a growing public health problem that particularly affects older adults. It is defined by the Portuguese National Health Service (NHS) as the subjective discrepancy between desired and actual social relationships, with a negative impact on quality of life [1].

The prevalence of loneliness in older adults has been documented in several international and national studies. In Portugal, the prevalence is estimated at 36% among individuals aged 65 years and over. It is associated with a higher risk of polypharmacy, depression, increased cardiovascular morbidity and mortality, and all-cause mortality [2-5]. Social prescribing (SP) is a holistic care model designed to improve health and well-being by addressing participants' non-medical, health-related social needs, such as social isolation and loneliness [6,7]. This approach involves referring patients to local, community-based, non-clinical resources and supports, often facilitated by trained link workers who help assess needs and ensure follow-through [6,8].

SP is emerging as a tool that integrates healthcare with community responses by formally referring individuals to leisure, cultural, sports, or volunteer activities, tailored to their interests and needs. The model, widely implemented in the United Kingdom, has demonstrated positive effects in reducing loneliness and unnecessary use of health services, as well as improving well-being and generating positive social returns [9,10].

While SP originated in the United Kingdom and is rapidly gaining global acceptance, the current evidence base on its effectiveness is limited by a lack of definitive causal studies, methodological challenges in initial program evaluations, and substantial variability in the outcomes measured across research projects [11]. The benefits of social prescribing are assessed using a wide range of instruments, many of which are patient-reported outcome measures focusing on psychosocial factors, such as well-being and mental health. The identified instruments fall across several core outcome areas, including physiological/clinical factors, life impact, resource use, and delivery of care [12]. In Portugal, pilot projects and local SP initiatives have emerged, recently framed by the creation of the National Social Prescription Network, which brings together multidisciplinary professionals and fosters institutional partnerships [13].

SP is met with high receptivity in Portugal, particularly among older adults, with over 75% agreeing that it would benefit their community and the national health system. Current evidence suggests that older adults rarely receive recommendations from health professionals for community activities and seldom present non-clinical complaints during appointments, revealing a gap in integrating non-medical approaches into primary care and emphasizing the importance of tailoring interventions to individual preferences and characteristics, promoting equity. Additionally, future studies that integrate SP into the healthcare system to support healthy aging are recommended [14]. Thus, there is a need for prospective studies to assess the impact of SP on the health of older adults and to evaluate the implementation of these models in the Portuguese health system.

This study describes the protocol for a study whose primary objective is to assess the impact of SP appointments on the perception of loneliness, well-being, quality of life, and psychological distress in an elderly population from three family health units (FHUs) of the local health unit (LHU) of Coimbra. The secondary objective focuses on identifying predictors of SP benefits and barriers, and on evaluating the intervention implementation process in the primary healthcare (PHC) setting. We aim to contribute to the development of similar projects at the regional level in a more structured and evidence-based manner, thereby facilitating their integration into the National Health System.

## Materials and methods

This study is a prospective, longitudinal, non-randomized controlled trial with an intervention and pre- and post-intervention evaluations, using a mixed-methods approach (quantitative and qualitative). The target population comprises older adults aged 65 years or over who use the following three FHUs in central Portugal: FHU 1 serves 2,598 elderly individuals (25.4% of its total population), with an elderly dependency index of 39.66%. FHU 2 serves 2,400 elderly individuals (27.4% of its total population), with an elderly dependency index of 44.54%. FHU 3 serves 1,963 elderly individuals (22.0% of its total population), with an elderly dependency index of 34.29%.

These units, located within a 5.5 km radius, serve a diverse and high-need population. This includes residents of deprived urban neighborhoods and municipal housing, a high proportion of migrants, and individuals in peripheral rural areas [15]. This study will include adults aged 65 years and older who will be assessed using the ALONE and Personal Well-Being Index (PWI) scales. Individuals scoring eight or higher on the ALONE scale or 69 or lower on the PWI scale will be selected for the intervention. Exclusion criteria include motor or cognitive impairments that limit understanding or participation, insufficient means of transportation, or unavailability to participate in the intervention.

Sample

Recruitment will target all individuals aged 65 years and older attending in-person appointments, whether with a nurse or a physician. At the time of appointment check-in or during the consultation itself, participants will be approached and invited to complete the initial recruitment questionnaire, which includes the ALONE and PWI scales. This questionnaire, along with the informed consent form, may be distributed by any trained member of the healthcare team, including clinical secretaries, nurses, physicians, or social workers, and subsequently analyzed by the research team. Based on the results of the ALONE and PWI scales, individuals who meet the preliminary criteria (ALONE ≥8 or PWI ≤69) will subsequently be invited to attend a dedicated social prescribing (SP) consultation, during which the full inclusion and exclusion criteria will be assessed. Those who meet the final eligibility criteria will then be enrolled in the study.

To ensure a robust evaluation of the intervention’s impact, participants will be divided into the following two groups: an intervention group receiving social prescribing consultations and community referrals, and a usual care comparator group. The latter will consist of individuals meeting the inclusion criteria who will continue with standard primary care and undergo the same consultations as the intervention group.

**Figure 1:**
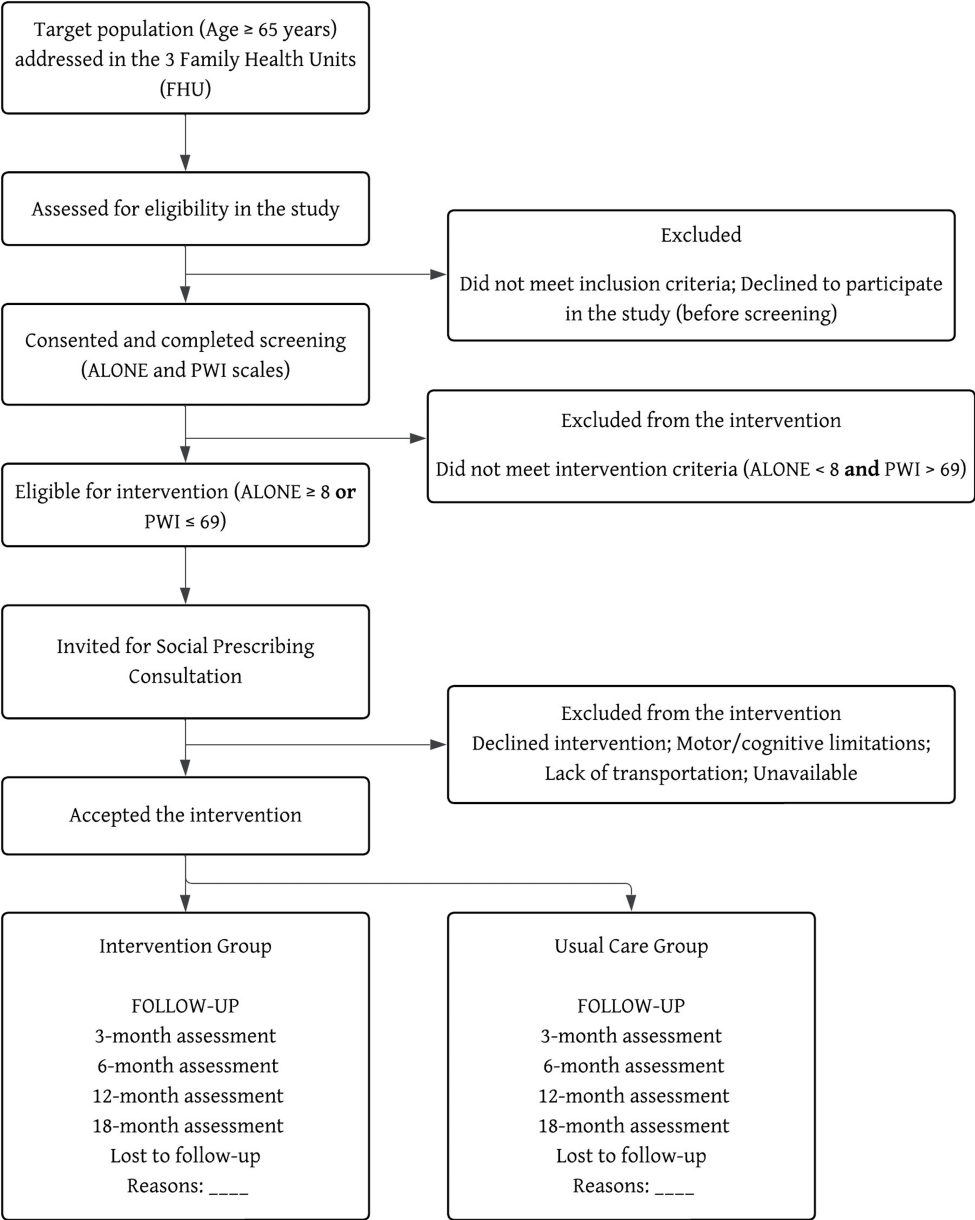
Flow chart of sample recruitment and follow-up.

Based on a previous study on the impact on quality of life, an initial sample size of 110 participants was calculated, assuming a significance level (α) of 5% and a statistical power (1-β) of 80% (β=20%) [16]. This estimate incorporates a conservative anticipated loss to follow-up of 50% over 18 months. Additionally, a usual care comparator group (n=55) will be recruited with a 2:1 allocation ratio. A lower attrition rate is anticipated for the control group due to the absence of the intervention's logistical demands. This total sample of 165 participants provides a robust basis for the planned longitudinal comparisons (Figure 1).

Intervention

The impact of SP appointments will be assessed using validated scales that evaluate the perception of loneliness (UCLA and ALONE scales), well-being (PWI scale), psychological distress (PHQ-4 scale), and quality of life (EQ-5D scale) in an elderly population from three FHUs of the local health unit (LHU) of Coimbra. All scales will be compiled into a single questionnaire comprising 39 items, which will be completed either on paper by the participants or via telephone interview.

During the initial SP appointment, in addition to the validated scales mentioned above, the individual’s profile will be characterized. This will be followed by a proposal for community activities tailored to the patient's physical condition, preferences, and social context.

The proposed activities will be selected from a catalog of local community resources, initially prepared and updated throughout the study, which includes walking football, water aerobics, creative workshops, choirs, volunteering, hiking, and cultural activities, among others. The SP consultation involves a collaborative assessment to match patient interests with the community catalog. Support is primarily motivational and informational; the professional provides details on how to access and register for the activities, though no direct financial support or transportation is provided by the study.

Participants will be reassessed in follow-up consultations (in person or by telephone) at three, six, 12, and 18 months to capture short-, medium-, and long-term effects, including the persistence of benefits post-engagement. In these assessments, the scales will be reapplied, and barriers to adherence will be identified.

Given the heterogeneity of the prescribed social activities (ranging from physical exercise to cultural workshops), defining a standardized minimum duration of participation is neither feasible nor appropriate within a person-centered model. Consequently, there is no minimum frequency or duration of activity required for a participant’s data to be included in the quantitative outcome analysis. The main objective is to evaluate the impact of the referral process and the opportunity for engagement. Adherence status (continuation or discontinuation of the activity) will be systematically recorded at each follow-up point; these data will primarily be used for qualitative assessment and for secondary analysis of barriers and facilitators (e.g., transport, cost, access) to inform future implementation strategies.

Data collection

Data will be collected longitudinally at multiple time points, including baseline and follow-up assessments at three, six, 12, and 18 months. At the baseline assessment, a comprehensive dataset will be collected. Initial screening and selection will be performed using the ALONE scale and the PWI. The ALONE scale (a five-item tool) is used primarily to screen for loneliness among older adults (aged 65+ years) by assessing social isolation, lack of companionship, and disconnection. In this scale, negative responses are assigned higher values; consequently, a higher total score reflects a greater risk of loneliness.

The PWI scale measures satisfaction across the following seven core life domains: standard of living, health, achievment in life, relationships, safety, community-connectedness, and future security. The scale consists of eight items, each rated from 0 to 10, where higher values indicate greater well-being. There are no formal clinical cut-offs, but scores below 50 points are generally interpreted as a signal of challenged homeostatic control and a higher risk of psychological distress.

Concurrently, sample characterization data will be gathered via a sociodemographic questionnaire (including gender, age, ethnicity, education, household and family support, etc.) and the family APGAR. Clinical data (multimorbidity, polypharmacy) will be extracted from patient records. At this baseline assessment, the primary outcome measures will be administered as follows: the UCLA Loneliness Scale, the EQ-5D, and the PHQ-4. The UCLA Loneliness Scale (three-item version) is an instrument used to assess subjective feelings of loneliness and social isolation. It consists of three items rated on a three-point Likert scale. Scores are summed to yield a total ranging from 3 to 9, with higher scores indicating greater loneliness; typically, a score of 6 or higher is used to classify an individual as lonely.

Psychological distress will be assessed using the PHQ-4, a four-item composite scale. Participants report the frequency of symptoms over the past two weeks on a four-point Likert scale. Total scores range from 0 to 12, with higher scores indicating greater psychological distress. Health-related quality of life will be measured using the EQ-5D-5L, which comprises two distinct sections. The first is a descriptive system assessing the following five dimensions: mobility, self-care, usual activities, pain/discomfort, and anxiety/depression, each with five levels of severity (from "no problems" to "extreme problems"). The second part is the EQ-VAS, a vertical visual analog scale on which participants rate their overall health from 0 (the worst health imaginable) to 100 (the best health imaginable). Higher scores in the descriptive system (before conversion to index values) indicate worse health states, whereas higher EQ-VAS scores reflect better self-reported health. At each follow-up point, the outcome measurement instruments (ALONE, UCLA, PWI, EQ-5D, and PHQ-4) will be readministered to monitor changes in outcomes over time.

In parallel, qualitative data will be collected from two perspectives. From the participants' perspective (including those who decline the intervention), reasons for refusal, perceived impact, barriers to adherence, and suggestions for improvement will be explored, using the Health Belief Model as a guiding framework. From the healthcare professionals' perspective, information on barriers, difficulties, and implementation suggestions will be collected using the RE-AIM framework. Table 1 details all instruments, variables, and the analytical focus for each data collection method.

**Table 1:** Description of scales and methods to be used for each study objective.

Phase/purpose of the evaluation	Instrument/variables	Focus of analysis
Initial screening and selection	ALONE scale [17]	Loneliness screening
	Personal Well-Being Index (PWI) [18]	Personal well-being
Impact measurement (quantitative)	UCLA Scale [19]	Loneliness
	EQ-5D Scale [20]	Quality of life
	PHQ-4 [21]	Psychological distress
Sample characterization (quantitative)	Sociodemographic questionnaire	Gender, age, ethnicity, marital status, household, family support, education, socioeconomic assessment, hobbies, social activities
	Family APGAR	Perception of family functioning
	Clinical data (case consultation)	Multimorbidity, polypharmacy (≥5 drugs)
Qualitative assessment (user perspective)	Health Belief Model	Reasons for refusing to participate; impact of the intervention; barriers to participation; suggestions for improvement
Qualitative assessment (professional perspective)	RE-AIM framework [22]	Barriers, difficulties, and suggestions for improving intervention (implementation)

Statistical analysis

Data analysis will be conducted using a mixed-methods approach. Descriptive statistics (means, medians, and proportions) will be used to characterize the sample, analyze adherence rates, and describe the reasons for non-adherence and the profile of individuals who decline the intervention.

Inferential statistics will be used to evaluate the intervention's impact. Changes in scores (ALONE, UCLA, PWI, EQ-5D, and PHQ-4) over time and comparisons between the intervention and usual care comparator groups will be conducted using mixed-effects models for repeated measures (MMRM) to account for longitudinal changes and baseline differences. Additionally, comparisons between intervention-adherent and non-adherent subgroups will be conducted using appropriate tests, such as t-tests, Mann-Whitney U tests, or chi-square tests, as appropriate for the data distribution. Regression models will be applied to evaluate correlations between sociodemographic/clinical variables and the evolution of loneliness, well-being, quality of life, and psychological distress, as well as to identify predictors of adherence.

Qualitative data on barriers and facilitators to implementation, collected from participants and healthcare professionals (using the RE-AIM framework), will be analyzed through categorical content analysis supported by specialized software. For all inferential statistical analyses, a significance level of p<0.05 will be adopted.

Ethical aspects

The study protocol will be submitted to the Ethics Committee of the Faculty of Medicine of Coimbra University and the Ethics Committee of the LHU Coimbra. Written informed consent will be obtained from all participants before their inclusion in the study. Data confidentiality will be ensured through anonymization and coding of all collected information. Permission will be obtained from the authors of the validated scales for their use in this project.

## Results

The primary expected outcome of this study is an improvement in participants’ well-being and mental health. It is hypothesized that the SP intervention will reduce loneliness (measured by the ALONE and UCLA scales) and psychological distress (PHQ-4), accompanied by a corresponding increase in quality of life (EQ-5D) and subjective well-being (PWI) over the 18-month follow-up period, and we hypothesize that these changes will be more robust in the SP intervention group, compared to the usual care group. Additionally, this project will allow a sociodemographic and clinical characterization of the participants, allowing group profiling and identification of specific barriers among those refusing intervention, providing crucial data on access equity and suggestions for improvement.

Regarding implementation, this study will generate a validated catalog of community resources, providing a practical and sustainable instrument for clinical practice. Qualitative analysis, based on the RE-AIM framework, is expected to identify contextual barriers and facilitators (such as professional workload and transport logistics) of the SP integration in primary healthcare in Portugal. In addition, longitudinal data collection and the use of mixed methods will grant transferable knowledge and practical recommendations to support legislators and health managers in the expansion of this intervention.

## Discussion

SP represents an innovative, increasingly recognized approach to address social determinants of health, such as loneliness among older adults. By bridging the gap between PHC and community resources, it offers a non-pharmacological, person-centered response to complex psychosocial needs that biomedical models often overlook [15].

International evidence, particularly from the United Kingdom, demonstrates the benefits of SP in reducing loneliness and improving quality of life [9,10]. However, methodological limitations and challenges with the generalizability of findings remain. Existing studies emphasize the need to include a broader range of community resources and more diverse populations, while integrating variables, such as ethnicity, educational attainment, and socioeconomic status, which are currently underreported in the literature. Furthermore, it is crucial to assess not only referral rates but also actual participation in prescribed activities, and expand the focus beyond physical exercise and arts-based interventions.

This field of research warrants more robust, longer-term designs incorporating control groups and mixed-methods to capture diverse perspectives, while also evaluating implementation factors, such as logistical barriers or delays, which directly impact adherence. Integration of behavioral change strategies and impact analysis of SP on primary healthcare professionals’ work could enhance the program’s efficacy and sustainability, enabling collaborative cost-effective models of support for data science [9].

The implementation of this protocol within a highly vulnerable population is a key strength of the pilot study. The three FHUs involved serve a combined elderly population of nearly 7,000 individuals. These units are characterized by significant elderly dependency indices, ranging from 34.29% to 44.54%. We anticipate individual-level barriers, such as motor or cognitive impairments, lack of transportation, and the potential cost of some community activities, as well as institutional barriers, such as professional workload. While these factors pose challenges to participation, the inclusion of a diverse sample that encompasses residents from deprived urban neighborhoods, municipal housing, migrant communities, and peripheral rural areas ensures that the intervention is tested against a broad spectrum of social realities. This diversity enhances the generalizability of our findings, as the protocol must adapt to varied sociogeographic contexts within a relatively small area.

This protocol aims to address some of these gaps through a prospective, mixed-methods pilot study conducted within Portuguese primary healthcare settings. By actively engaging patients, healthcare professionals, and community organizations, it aims to produce evidence on the impact, acceptability, and feasibility of SP in a regional context, contributing to the development of sustainable, integrated practices that promote well-being and social cohesion.

## Conclusions

The results of this longitudinal prospective pilot study will provide preliminary evidence on the associations between social prescribing and changes in perceived loneliness, well-being, and psychological distress among older adults, along with the identification of barriers and facilitators to adherence to the proposed activities. Given its pilot design, the study focuses on documenting within-person changes over time and evaluating implementation outcomes, aiming to generate evidence on the benefits of SP and to optimize its implementation and sustainable integration into the Portuguese National Health Service (NHS).

Thus, the project aligns with national and international efforts to strengthen the role of PHC in addressing the social determinants of health, bringing medicine closer to the community, promoting active aging, social inclusion, and well-being, and consolidating SP as a strategic tool for more humane and equitable health systems.

## References

[1] SNS SNS (2025). A solidão e o isolamento social. Serviço Nacional de Saúde.

[2] Rocha-Vieira C, Oliveira G, Couto L, Santos P (2019). Impact of loneliness in the elderly in health care: a cross-sectional study in an urban region of Portugal. Fam Med Prim Care Rev.

[3] Martins IM, Almeida IM, Carreira IF (2023). A Solidão na velhice e a intervenção social em Portugal. RIAGE.

[4] Valtorta NK, Kanaan M, Gilbody S, Ronzi S, Hanratty B (2016). Loneliness and social isolation as risk factors for coronary heart disease and stroke: systematic review and meta-analysis of longitudinal observational studies. Heart.

[5] Cacioppo JT, Cacioppo S (2014). Social relationships and health: the toxic effects of perceived social isolation. Soc Personal Psychol Compass.

[6] Yu C, Lail S, Allison S (2024). Social prescribing needs and priorities of older adults in Canada: a qualitative analysis. Health Promot Chronic Dis Prev Can.

[7] Hough K, Kotwal AA, Boyd C, Tha SH, Perissinotto C (2023). What are "social prescriptions" and how should they be integrated into care plans?. AMA J Ethics.

[8] Hamilton-West K, Milne A, Hotham S (2020). New horizons in supporting older people's health and wellbeing: is social prescribing a way forward?. Age Ageing.

[9] Percival A, Newton C, Mulligan K, Petrella RJ, Ashe MC (2022). Systematic review of social prescribing and older adults: where to from here?. Fam Med Community Health.

[10] Sadio R, Henriques A, Nogueira P, Costa A (2024). Social prescription for the elderly: a community-based scoping review. Prim Health Care Res Dev.

[11] Lee H, Koh SB, Jo HS (2023). Global trends in social prescribing: web-based crawling approach. J Med Internet Res.

[12] Ashe MC, Santos IK, Alfares H, Chudyk AM, Esfandiari E (2024). Evidence synthesis - outcomes and instruments used in social prescribing: a modified umbrella review. Health Promot Chronic Dis Prev Can.

[13] (2024). NOVA: prescrição social Portugal. https://www.ensp.unl.pt/knowledge-centers/prescricaosocial/.

[14] Costa A, Henriques J, Alarcão V (2024). Social prescribing for older adults in mainland Portugal: perceptions and future prospects. Prev Med Rep.

[15] (2025). Bilhete de identidade dos cuidados de saúde primários (BI-CSP). https://bicsp.min-saude.pt/pt/Paginas/default.aspx.

[16] Dickens AP, Richards SH, Hawton A (2011). An evaluation of the effectiveness of a community mentoring service for socially isolated older people: a controlled trial. BMC Public Health.

[17] Guerra SR, Sousa L, Silva SL, Martins MR, Tavares JP (2024). ALONE Scale for Portuguese Older Adults: translation, adaptation, content validity, and face validity. Rev Enferm Ref.

[18] Pais-Ribeiro Pais-Ribeiro, J. and Cummins, R R (2008). O bem-estar pessoal: estudo de validação da versão portuguesa da escala. https://www.researchgate.net/publication/262270384_O_bem-estar_pessoal_estudo_de_validacao_da_versao_portuguesa_da_escala#fullTextFileContent.

[19] Pocinho M, Farate C, Dias CA (2010). Validação Psicométrica da Escala UCLA-Loneliness para Idosos Portugueses. Interações Soc Novas Modernidades.

[20] Ferreira PL, Ferreira LN, Pereira LN (2013). Contribution for the validation of the Portuguese version of EQ-5D. Acta Med Port.

[21] Kroenke K, Spitzer RL, Williams JB, Löwe B (2009). An ultra-brief screening scale for anxiety and depression: the PHQ-4. Psychosomatics.

[22] Glasgow RE, Vogt TM, Boles SM (1999). Evaluating the public health impact of health promotion interventions: the RE-AIM framework. Am J Public Health.

